# Prevalence of six respiratory pathogens among children in Chengdu, China: a multiplex PCR-based detection study

**DOI:** 10.3389/fcimb.2025.1690701

**Published:** 2025-12-01

**Authors:** Chenggui Liu, Jiuda Li, Yao Deng, Qin Wang, Xuemei Wang, Shuzhe Yang, Weijun He, Zhiyong Liao

**Affiliations:** 1 Department of Performance Appraisal Management, Chengdu Women’s and Children’s Central Hospital, School of Medicine, University of Electronic Science and Technology of China, Chengdu, China; 2 Department of Clinical Laboratory, Chengdu Women’s and Children’s Central Hospital, School of Medicine, University of Electronic Science and Technology of China, Chengdu, China; 3 Department of Clinical Laboratory, Sichuan Province Orthopedic Hospital, Chengdu, China

**Keywords:** influenza A virus, influenza B virus, respiratory syncytial virus, adenovirus, rhinovirus, *Mycoplasma pneumoniae*, respiratory tract infections, children

## Abstract

**Background:**

Respiratory tract infections among children are commonly caused by several pathogens, and their prevalence varies across different groups. The goal of this study is to investigate the prevalence of Influenza A virus (Flu A), Influenza B virus (Flu B), respiratory syncytial virus (RSV), Adenovirus (ADV), Rhinovirus (RV), and *Mycoplasma pneumoniae* (MP) among children in different groups in Chengdu and analyze their differences.

**Methods:**

This retrospective cross-sectional study included 39,190 children, with 21,847 males and 17,343 females. All respiratory specimens from the participants were tested for Flu A, Flu B, RSV, ADV, RV, and MP using multiplex PCR.

**Results:**

The overall prevalence of single infection, double co-infection, and triple co-infection was 48.19%, 7.09%, and 0.25%, respectively. The pathogen-specific prevalence from highest to lowest was RV (21.43%), ADV (16.69%), MP (11.73%), RSV (8.12%), Flu A (3.78%), and Flu B (1.37%). Significant differences were observed in the prevalence of the six pathogens across all six age groups (all *p* < 0.001). The prevalence of Flu A, Flu B, ADV, and MP was highest in school-aged children and lowest in newborns; RSV prevalence peaked in infants and was lowest in adolescents; RV was most prevalent in toddlers and least in newborns. The prevalence of Flu A, Flu B, and RSV was significantly higher in spring/winter than in summer/autumn (*p* < 0.001). MP and RV prevalence was significantly higher in spring/summer than in autumn/winter (*p* < 0.001), while ADV prevalence was significantly higher in autumn/summer than in winter/spring (*p* < 0.001). Among the five clinical diagnosis groups, Flu A and Flu B prevalence was highest in SRLT and lowest in CRDs; RSV and MP peaked in ALRTIs and bottomed in AURTIs; RV was highest in CRDs and lowest in AURTIs; ADV was highest in AURTIs and lowest in CRDs.

**Conclusions:**

Over half of the children were infected with at least one of the six respiratory pathogens, with RV, ADV, and MP being predominant. While co-infections were less common than single infections, they still occurred, with double co-infections being the main form. Notably, the prevalence varied significantly by age, season, and clinical diagnosis. These findings may offer useful references for developing targeted prevention and control strategies.

## Introduction

Respiratory tract infections (RTIs) represent a severe and widespread health issue, particularly among the pediatric population globally. These infections are highly prevalent and rank among the most common respiratory diseases affecting children worldwide. Currently, RTIs pose a significant and far-reaching challenge to public health, primarily attributed to their high morbidity and mortality rates (Yang et al., 2022; Xu et al., 2025). A wide range of pathogens can cause RTIs, including viruses, bacteria, atypical pathogens, fungi, and parasites (Niederman and Torres, 2022; Georgakopoulou et al., 2024). Among these, viruses are the predominant infectious agents. Influenza A virus (Flu A), Influenza B virus (Flu B), respiratory syncytial virus (RSV), Adenovirus (ADV), and Rhinovirus (RV) are the most common respiratory viruses that cause RTIs (Lin et al., 2020; Cilloniz et al., 2022). Furthermore, the emergence of coronaviruses—particularly the global spread of severe acute respiratory syndrome coronavirus type 2 (SARS-CoV-2), the causative agent of COVID-19—has underscored their significant role in the spectrum of respiratory pathogens and the critical need for their surveillance (Tabibzadeh et al., 2020). Moreover, co-infections of SARS-CoV-2 with other respiratory viruses, such as Flu A, Flu B, RSV, and RV, are not uncommon, and such viral co-infections may be associated with more severe clinical outcomes (Lai et al., 2020; Eisen et al., 2021; Sadeh Tehrani et al., 2024). In addition, with the advancement of pathogen detection technology, especially the widespread application of nucleic acid amplification tests (NAATs) in clinical practice, the role of atypical pathogens in RTIs, such as *Mycoplasma pneumoniae* (MP), has attracted increasing attention (Miyashita, 2022; Liu et al., 2023).

The pathogens causing RTIs exhibit remarkable adaptability and the ability to infiltrate both the upper respiratory tract and lower respiratory tract. When the upper respiratory tract is invaded, these pathogens may give rise to a range of diseases such as pharyngitis, laryngitis, and sinusitis. Viral agents like Flu A, Flu B, RSV, ADV, and RV, are among the most common triggers for such upper RTIs (Clementi et al., 2021). In contrast, when the lower respiratory tract is affected by these pathogens, especially in vulnerable populations such as infants, young children, or immunocompromised individuals, it can trigger more severe conditions. These include tracheitis, bronchitis, bronchiolitis, and pneumonia (Lowe, 2022; Hong et al., 2024). Flu A and Flu B are well-known for their biphasic tropism. Infection with Flu A and/or Flu B may cause mild respiratory symptoms, which are initially confined to the URT. Typical manifestations include fever, sore throat, rhinitis, cough, fatigue, and headache and then progress to cause more significant damage to the LRT, potentially leading to severe pneumonia (Mifsud et al., 2021). MP can also cause URT and/or LRT infections, typically presenting with a persistent cough. In immunocompromised children and other susceptible populations, it may lead to more serious respiratory complications and extrapulmonary manifestations, including involvement of the skin, hematologic system, cardiovascular system, musculoskeletal system, and nervous system (Loconsole et al., 2021).

There are several methods for diagnosing pathogens causing RTIs, including microscopy, culture, immunoassays (for antigen or antibody detection), and NAATs such as traditional polymerase chain reaction (PCR), nested PCR, real-time quantitative PCR, and multiplex PCR. Microscopy with Gram staining provides rapid preliminary assessment for certain bacterial infections, but its sensitivity is significantly lower than other methods and it is ineffective for cell-wall-deficient pathogens like MP. Although MP can be cultured for diagnosis and drug susceptibility testing, this method is not routinely used as a preferred approach in clinical practice due to its technically demanding nature, prolonged turnaround time, low yield, and associated challenges (Kumar and Kumar, 2023). For viral RTIs, both microscopy and culture face substantial limitations: conventional light microscopy cannot resolve viruses, electron microscopy is impractical for routine use, and viral culture requires specialized cell lines, stringent laboratory conditions, extended incubation periods, and may still miss fastidious or uncommon strains, resulting in poor sensitivity and limited clinical utility.

Immunoassays for antigens or antibodies have diagnostic value in the detection of pathogens causing RTIs. For pathogens such as RSV, Flu A, and Flu B, antigen detection is faster, easier to perform, and less costly, which makes it suitable for patients with high viral loads. However, these tests have lower sensitivity and specificity, leading to false-negative results (especially at low viral loads) and false-positive results due to potential cross-reactivity (Li et al., 2024; Murphy et al., 2024). In contrast, serological antibody tests (IgM and/or IgG) are applicable for diagnosing pathogens causing RTIs, including RSV, Flu A, Flu B, ADV, and atypical pathogens such as MP (Liu et al., 2023). These tests are particularly useful for detecting specific IgM, which supports serological diagnosis (Liu et al., 2023; Tian et al., 2023). However, they have several limitations, including a delayed antibody response window that may lead to early false negatives, an inability to distinguish acute from past infections solely based on IgG positivity, and a need for clinical correlation for accurate result interpretation. Additionally, serology testing cannot track the dynamics of an infection or identify individuals immune to influenza-like illnesses (Toczek-Kubicka et al., 2022).

Metagenomic next-generation sequencing (mNGS) can also be used to detect pathogens causing RTIs. However, mNGS is both complex and expensive, and it may face challenges such as sample contamination risks and difficult result interpretation in clinical applications, which to some extent limits its widespread adoption in emergency or primary healthcare settings (Yin et al., 2024). By contrast, clinical molecular diagnostic techniques based on PCR, leveraging the dual advantages of high sensitivity and specificity, have demonstrated significant analytical and clinical benefits in the detection of pathogens causing RTIs, gradually replacing traditional identification techniques in many clinical scenarios. Besides high sensitivity and specificity, multiplex PCR panels also offer several advantages, including small sample requirement, rapid detection, etc. They can simultaneously detect multiple pathogens, providing accurate etiological evidence for mixed infections to optimize clinical decision-making (Clark et al., 2023; Azizian et al., 2025).

In this study, a multiplex PCR panel was employed to detect Flu A, Flu B, RSV, ADV, RV, and MP in respiratory specimens from children in Chengdu, China. The objective is to investigate the prevalence of these six respiratory pathogens (including patterns of single and co-infections) among children in different age groups, across seasons, and among clinical diagnosis subgroups in this region, and to analyze differences in their distribution, thereby providing a basis for developing targeted prevention and control strategies for pediatric RTIs.

## Materials and methods

### Study participants

**Figure 1:**
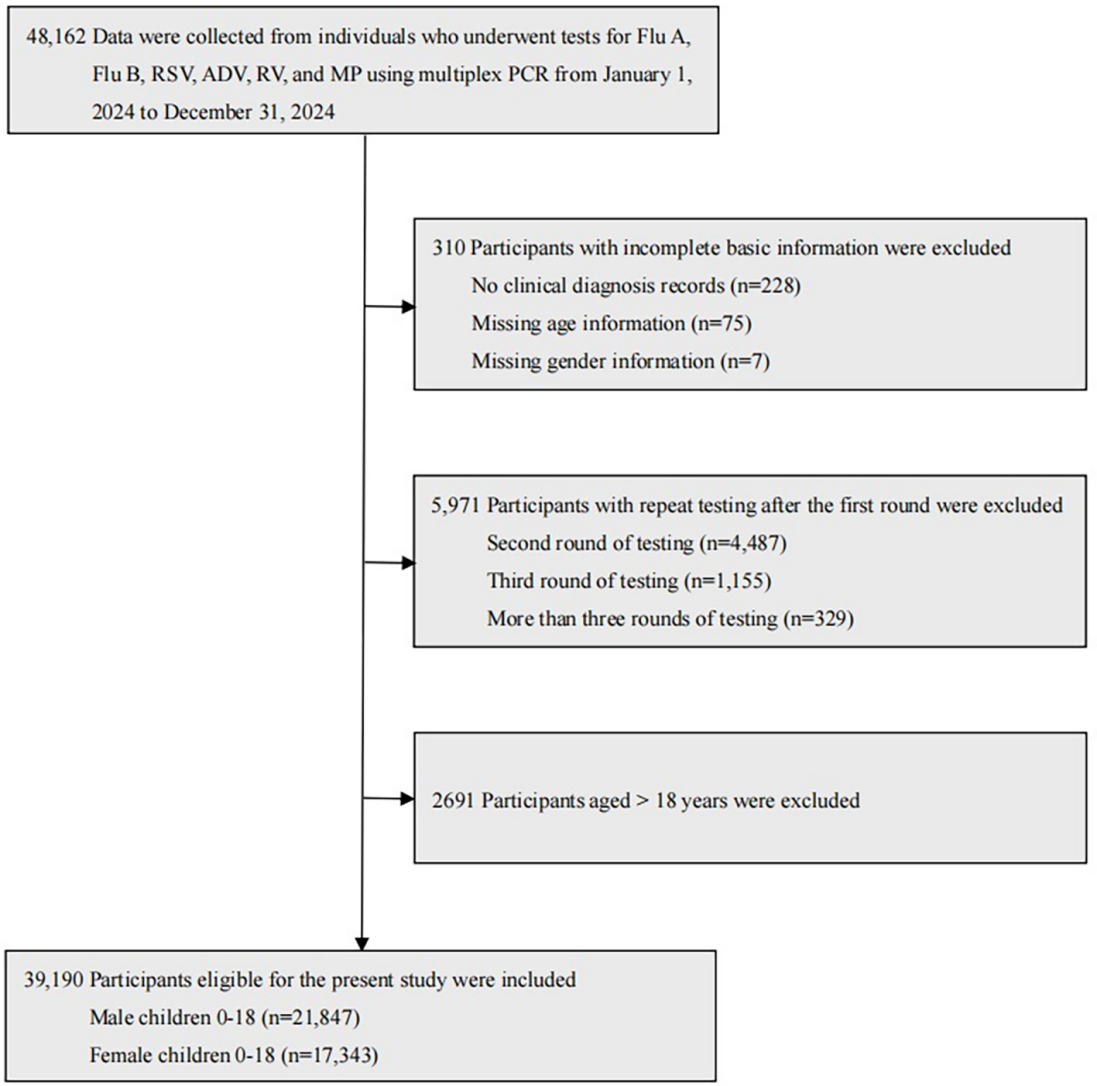
Selection of male and female child participants aged 0–18 years.

A retrospective cross-sectional study was conducted at Chengdu Women’s and Children’s Central Hospital, School of Medicine, University of Electronic Science and Technology of China, covering the period from January 1, 2024, to December 31, 2024. A total of 48,162 individuals underwent tests for Flu A, Flu B, RSV, ADV, RV, and MP using multiplex PCR on pharyngeal swab specimens. Among them, 39,190 eligible child participants were included in the present study, comprising 21,847 male participants aged 0–18 years (mean age: 3.29 ± 3.02 years) and 17,343 female participants aged 0–18 years (mean age: 3.59 ± 3.10 years). Specifically, children were divided into six age groups: 1,451 newborns aged 0 to 28 days, 7,222 infants aged 29 days to 1 year, 8,599 toddlers aged 1 to 3 years, 12,778 preschoolers aged 3 to 6 years, 8,491 school-aged children aged 6 to 12 years, 649 adolescents aged 12 to 18 years. The child participant selection process is described in detail in **Figure 1**.

The exclusion criteria were as follows: (a) Participants with incomplete basic information, including lack of clinical diagnosis records, or missing age or gender information. (b) Participants with repeated tests conducted within 28 days after the first round. (c) Participants aged over 18 years.

According to the medical settings, 39,190 eligible child participants were divided into outpatients (n=7,752) and inpatients (n=31,438). Based on their clinical diagnoses, participants were categorized into five groups: acute lower respiratory tract infections (ALRTIs), acute upper respiratory tract infections (AURTIs), chronic respiratory diseases (CRDs), non-respiratory diseases (NRDs), and self-requested laboratory testing (SRLT). For participants with multiple clinical diagnoses, the first diagnosis recorded in medical records was selected as the diagnostic basis for this study.

1. ALRTIs include pneumonia, acute tracheitis, acute bronchitis, acute tracheobronchitis, acute laryngotracheitis, acute laryngotracheobronchitis, and acute bronchiolitis.
2. AURTIs include acute pharyngitis, acute laryngitis, acute tonsillitis, acute rhinitis, acute sinusitis, acute epiglottitis, and the common cold.
3. CRDs include chronic pharyngitis, chronic rhinitis, chronic sinusitis, chronic bronchitis, bronchiectasis, bronchial asthma, and pulmonary tuberculosis.
4. NRDs include Henoch-Schönlein purpura (allergic purpura), thrombocytopenic purpura, leukemia, hemophilia, infectious mononucleosis, hypothyroidism, hyperthyroidism, type 1 diabetes, diarrhea, acute gastroenteritis, acute appendicitis, acute parotitis, acute lymphadenitis, acute urinary tract infection, sepsis, epilepsy, myocarditis, Kawasaki disease, acute conjunctivitis, neonatal hyperbilirubinemia, acute urticaria, constipation etc.
5. SRLT refers to self-requested laboratory testing by participants or their legal guardians without prior physician assessment or recommendation, regardless of subjective health concerns.

### Specimen collection

Pharyngeal swab sampling for children is carried out by clinicians or nurses following these procedures: (1) newborns and infants: Have a caregiver securely hold the child to stabilize the head and body. If necessary, clean the oral cavity with sterile saline. Use a small sterile swab, gently insert it into the pharynx, stay for a few seconds, rotate softly 1–2 turns, and remove the swab. (2) toddlers and preschoolers: Ask a caregiver to assist in holding the child to prevent movement. Instruct or guide the child to open the mouth; clean the oral area with sterile saline if needed. Insert a sterile swab into the pharynx, stay briefly, rotate gently 1–2 turns, and withdraw the swab. (3) school-aged children and adolescents: Instruct the child to open the mouth and say “ah” to expose the pharynx. Clean the oral region with sterile saline if necessary. Insert a sterile swab into the pharynx, stay for a few seconds, rotate firmly 1–2 turns, and remove the swab. All specimens were tested within 24 hours.

### Multiplex PCR assay

Six respiratory pathogens (Flu A, Flu B, RSV, ADV, RV, and MP) were detected using a multiplex PCR nucleic acid diagnostic kit (Sansure Biotech Inc., Changsha, China). The standard operating procedures (SOP) were as follows:

#### Preparation of reagents

Remove all kit components from storage and allow them to equilibrate to room temperature. Briefly vortex each component and set aside for use. Prepare the negative control containing internal control by adding 10 μL of internal control to every 200 μL of negative control, mix thoroughly, and briefly centrifuge. Based on the number of test specimens plus positive and negative controls, prepare PCR Master Mix A by combining 43.5 μL of PCR Mix A and 1.5 μL of Enzyme Mix per reaction; mix thoroughly and briefly centrifuge. Similarly, prepare PCR Master Mix B using PCR Mix B and Enzyme Mix in the same proportions; mix thoroughly and briefly centrifuge.

#### Specimen processing and loading

Add 200 μL each of the test specimen, negative control, and positive control into separate 1.5 mL centrifuge tubes. Extract nucleic acids using the Multi-type Sample DNA/RNA Extraction-Purification Kit (Magnetic Bead Method) manufactured by Sansure Biotech Inc., following the product’s instructions. Add 5 μL of each extracted nucleic acid (from test specimens, positive control, and negative control) into corresponding 0.2 mL PCR reaction tubes, then add 45 μL of PCR Master Mix A to each tube and seal the tube caps. Similarly, add 5 μL of the extracted nucleic acids into a separate set of 0.2 mL PCR reaction tubes, add 45 μL of PCR Master Mix B to each, and seal the tube caps.

#### PCR amplification

Use the MA-6000 Real-Time Quantitative Thermal Cycler (Suzhou Molarray Co., Ltd., Suzhou, China). Place the PCR reaction tubes into the sample wells of the amplification instrument, arranging the positive control, negative control, and unknown specimens in sequence, and input the specimen information. For reactions with PCR Master Mix A, select the FAM channel (Reporter: FAM, Quencher: None) for Flu A detection, HEX or VIC channel (Reporter: HEX/VIC, Quencher: None) for Flu B, CY5 channel (Reporter: CY5, Quencher: None) for RSV, and ROX channel (Reporter: ROX, Quencher: None) for internal control; set the reaction volume to 50 μL. For reactions with PCR Master Mix B, select the FAM channel (Reporter: FAM, Quencher: None) for ADV, HEX or VIC channel (Reporter: HEX/VIC, Quencher: None) for RV, CY5 channel (Reporter: CY5, Quencher: None) for MP, and ROX channel (Reporter: ROX, Quencher: None) for internal control; set the reaction volume to 50 μL. Set the cycling parameters as follows: Step 1 (reverse transcription) at 50 °C for 30 min (1 cycle); Step 2 (pre-denaturation) at 95 °C for 1 min (1 cycle); Step 3 (denaturation) at 95 °C for 15 sec and Step 4 (annealing, extension, and fluorescence collection) at 60 °C for 30 sec (45 cycles); Step 5 (optional instrument cooling) at 25 °C for 10 sec (1 cycle).

#### Result interpretation

A specimen was considered positive for a respiratory pathogen if the corresponding detection channel (within its assigned Master Mix reaction) exhibited a typical S-shaped amplification curve with a cycle threshold (Ct) value ≤ 40. Specifically, positivity for Flu A, Flu B, or RSV was determined in the Master Mix A reaction (FAM, HEX/VIC, and CY5 channels, respectively), while positivity for ADV, RV, or MP was determined in the Master Mix B reaction (FAM, HEX/VIC, and CY5 channels, respectively). A specimen was deemed negative for a pathogen if no amplification curve (no Ct value detected) or a Ct value > 40 was observed in its specific detection channel. For validity verification via the ROX internal control channel: no specific requirement was imposed on the internal control result for positive specimens, while negative specimens (no target pathogen detected) were required to have a positive internal control result (Ct ≤ 40) to confirm valid specimen processing and assay performance. If the internal control for a negative specimen showed a Ct value > 40 or no Ct value was detected, the test result was classified as invalid, and relevant factors (e.g., specimen quality, nucleic acid extraction efficiency, or reagent integrity) were investigated before recollecting and retesting the specimen.

### Statistical analysis

SPSS software version 19.0 (SPSS, Inc., Chicago, IL, USA) was used for all statistical analyses. The prevalence of Flu A, Flu B, RSV, ADV, RV, and MP was expressed as percentages, with differences analyzed using the Chi-Square test (*χ*^2^). Initially, univariate logistic regression was applied to assess associations between positivity for each pathogen and relevant variables, including gender, age, medical settings, seasons, and clinical diagnoses. Variables yielding a *p*-value < 0.25 in univariate analysis were deemed potentially relevant and included in subsequent analyses. Multivariate logistic regression was then performed to determine the independent effects of these variables on pathogen positivity, employing the Stepwise Forward Wald method to select the most impactful variables for the final model while adjusting for potential confounders. Odds ratios (OR) and their 95% confidence intervals (95% CI) were computed to quantify the strength and direction of associations, enabling the identification of variables that independently influence outcomes and enhancing the robustness of associations between predictors and pathogen positivity. A *p*-value < 0.05 was defined as statistically significant.

## Results

### Prevalence of six respiratory pathogens among children

More than half (55.53%) of the children were infected with at least one of the six respiratory pathogens. The overall prevalence of single infection, double co-infection, and triple co-infection was 48.19%, 7.09%, and 0.25%, respectively, with no quadruple, quintuple, or sextuple co-infections observed. The pathogen-specific prevalence from the highest to the lowest was as follows: RV (21.43%), ADV (16.69%), MP (11.73%), RSV (8.12%), Flu A (3.78%), and Flu B (1.37%) (*χ*^2^ = 12,221.187, *p* < 0.001). For single infections, the pathogens with the highest prevalence were RV (16.32%), ADV (12.98%), and MP (8.76%), while those with the lowest prevalence were Flu B (1.01%), Flu A (3.00%), and RSV (6.12%). For double co-infections, the combinations with the highest prevalence were ADV+RV (2.18%), RV+MP (1.48%), and RSV+RV (0.94%), whereas those with the lowest prevalence were Flu A+Flu B (0.01%), Flu B+ADV (0.05%), and Flu B+RV (0.05%). For triple co-infections, ADV+RV+MP (0.08%), RSV+ADV+RV (0.05%), and RSV+RV+MP (0.03%) showed the highest prevalence, in contrast to the three combinations including Flu A+Flu B+ADV, Flu A+RSV+MP, and Flu B+RSV+ADV, for which no cases were detected. The prevalence of single infection and multiple co-infections with the six pathogens among male and female children and the prevalence of specific double co-infections and triple co-infections with these six pathogens in 39,190 children are reported in **Table 1** and **Table 2**, respectively.

**Table 1:** Prevalence of single infections and multiple co-infections with Flu A, Flu B, RSV, ADV, RV and MP in male and female children.

Infection types	Total		Males		Females		*χ* ^2^	*p*-value
	n	Prevalence (%)	n	Prevalence (%)	n	Prevalence (%)		
Overall single infection	39,190	48.19	21,847	48.75	17,343	47.48	6.241	0.012
Flu A single infection	39,190	3.00	21,847	2.98	17,343	3.03	0.090	0.765
Flu B single infection	39,190	1.01	21,847	1.13	17,343	0.87	6.286	0.012
RSV single infection	39,190	6.12	21,847	6.25	17,343	5.96	1.432	0.231
ADV single infection	39,190	12.98	21,847	12.98	17,343	12.98	0.000	0.996
RV single infection	39,190	16.32	21,847	16.94	17,343	15.55	13.578	< 0.001
MP single infection	39,190	8.76	21,847	8.49	17,343	9.10	4.539	0.033
Overall double co-infection	39,190	7.09	21,847	7.18	17,343	6.97	0.624	0.430
Overall triple co-infection	39,190	0.25	21,847	0.28	17,343	0.21	1.682	0.195
Overall quadruple co-infection	39,190	0	21,847	0	17,343	0	/	/
Overall quintuple co-infection	39,190	0	21,847	0	17,343	0	/	/
Overall sextuple co-infection	39,190	0	21,847	0	17,343	0	/	/

**Table 2:** Prevalence of specific double co-infections and triple co-infections with Flu A, Flu B, RSV, ADV, RV, and MP in 39,190 children.

Double co-infections			Triple co-infections		
Combination types	Infected cases	Prevalence (%)	Combination types	Infected cases	Prevalence (%)
Flu A+Flu B	3	0.008	Flu A+Flu B+RSV	1	0.003
Flu A+RSV	44	0.112	Flu A+Flu B+ADV	0	0.000
Flu A+ADV	92	0.235	Flu A+Flu B+RV	1	0.003
Flu A+RV	95	0.242	Flu A+Flu B+MP	1	0.003
Flu A+MP	53	0.135	Flu A+RSV+ADV	2	0.005
Flu B+RSV	25	0.064	Flu A+RSV+RV	1	0.003
Flu B+ADV	20	0.051	Flu A+RSV+MP	0	0.000
Flu B+RV	20	0.051	Flu A+ADV+RV	8	0.020
Flu B+MP	61	0.156	Flu A+ADV+MP	2	0.005
RSV+ADV	149	0.380	Flu A+RV+MP	3	0.008
RSV+RV	370	0.944	Flu B+RSV+ADV	0	0.000
RSV+MP	148	0.378	Flu B+RSV+RV	3	0.008
ADV+RV	856	2.184	Flu B+RSV+MP	1	0.003
ADV+MP	262	0.669	Flu B+ADV+RV	1	0.003
RV+MP	579	1.477	Flu B+ADV+MP	2	0.005
			Flu B+RV+MP	1	0.003
			RSV+ADV+RV	20	0.051
			RSV+ADV+MP	6	0.015
			RSV+RV+MP	13	0.033
			ADV+RV+MP	32	0.082
Total	2,777	7.086	Total	98	0.250

**Figure 2:**
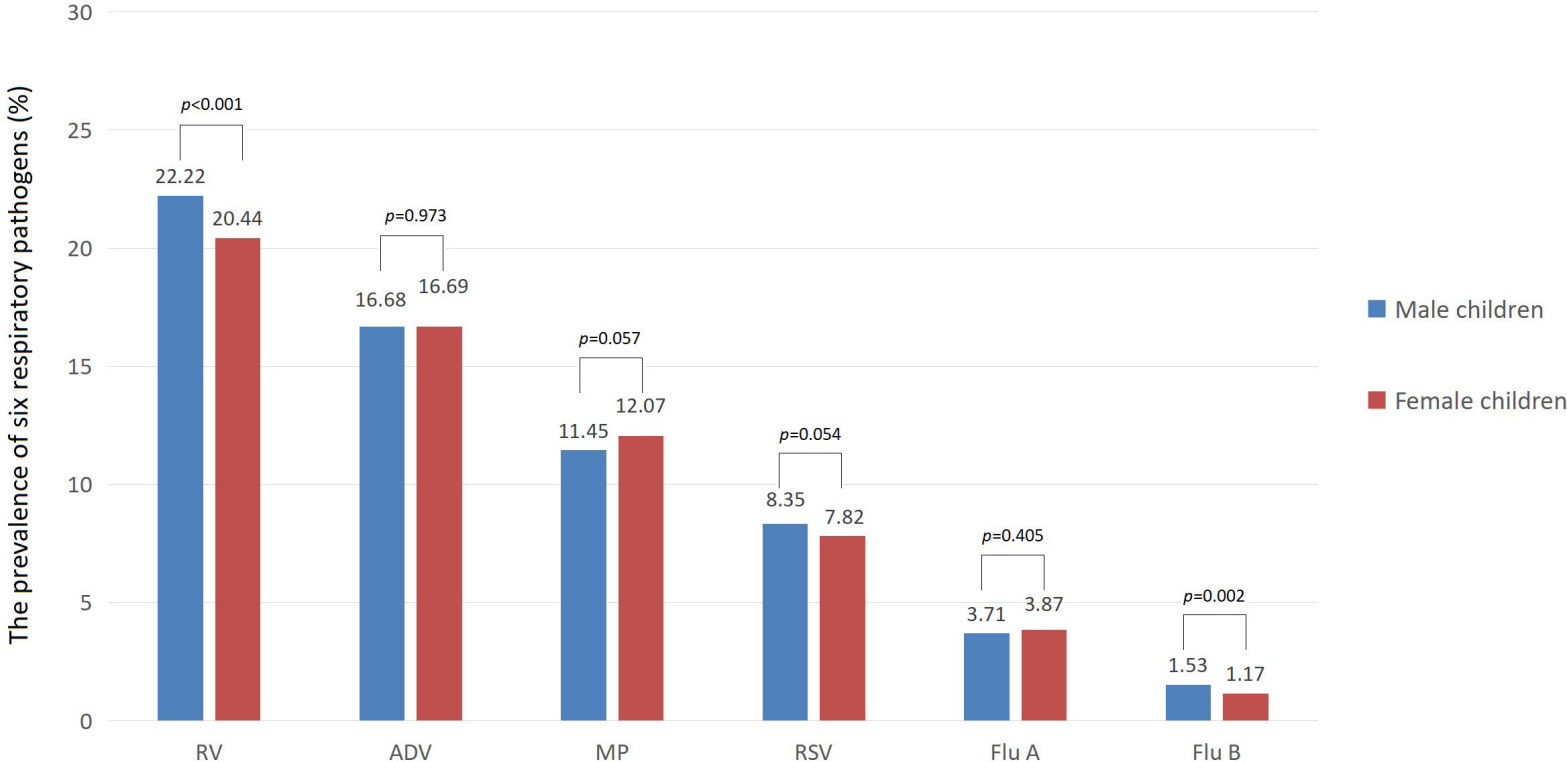
Prevalence of six respiratory pathogens among both males and females children (%).

In terms of gender, the prevalence of Flu B and RV was slightly higher among male children than among female children (1.53% vs 1.17% for Flu B, 22.22% vs 20.44% for RV, both *p* < 0.01). However, there was no significant difference in the prevalence of Flu A, RSV, ADV, and MP between male and female children (all *p*>0.05). In terms of medical settings, the prevalence of Flu A, Flu B, and ADV among children was higher in outpatient than in inpatient (7.68% vs 2.82% for Flu A, 2.22% vs 1.16% for Flu B, 30.71% vs 13.23% for ADV, all *p* < 0.001). However, the prevalence of RSV, RV, and MP among children was lower in outpatient than in inpatient (5.90% vs 8.66% for RSV, 15.83% vs 22.82% for RV, 9.57% vs 12.26% for MP, all *p* < 0.001). The prevalence of six respiratory pathogens among male and female children is shown in **Figure 2**.

### Comparison of the prevalence of six respiratory pathogens in different age groups among children

The prevalence of Flu A, Flu B, ADV, and MP was the highest in school-aged children (5.05% for Flu A, 1.96% for Flu B, 24.00% for ADV and 20.85% for MP) and the lowest in newborns (0.41% for Flu A, 0.48% for Flu B, 2.96% for ADV and 1.31% for MP), with the remaining age groups—infants, toddlers, preschoolers, and adolescents—showing intermediate values. Specifically, for Flu A, the prevalence decreased in the following order: toddlers (4.04%), preschoolers (3.97%), adolescents (3.54%), and infants (2.34%). For Flu B, the order of decreasing prevalence was: preschoolers (1.32%), infants (1.29%), toddlers (1.12%), and adolescents (0.92%). For ADV, the decreasing order was: preschoolers (22.77%), toddlers (13.39%), adolescents (8.63%), and infants (4.74%). For MP, the order of decreasing prevalence was: preschoolers (13.20%), adolescents (12.33%), toddlers (8.41%), and infants (4.39%). There were significant differences in prevalence among these six age groups (all *p* < 0.001).

The prevalence of RSV was the highest in infants (15.31%) and the lowest in adolescents (2.31%), with toddlers (11.33%), newborns (7.24%), preschoolers (6.04%), and school-aged children (2.46%) showing intermediate values in descending order; there were significant differences in prevalence among these six age groups (*p* < 0.001). The prevalence of RV was the highest in toddlers (26.20%) and the lowest in newborns (9.30%), with preschoolers (23.99%), infants (20.33%), school-aged children (16.31%), and adolescents (14.48%) showing intermediate values in descending order; there were significant differences in prevalence among these six age groups (*p* < 0.001). The comparison of the prevalence of six respiratory pathogens in different age groups among children is reported in **Table 3**.

**Table 3:** Comparison of prevalence of Flu A, Flu B, RSV, ADV, RV and MP in different age groups among children.

Age groups	Flu A		Flu B		RSV		ADV		RV		MP	
	n	Prevalence (%)	n	Prevalence (%)	n	Prevalence (%)	n	Prevalence (%)	n	Prevalence (%)	n	Prevalence (%)
Newborns	1,451	0.41	1,451	0.48	1,451	7.24	1,451	2.96	1,451	9.30	1,469	1.31
Infants	7,222	2.34 ^a^	7,222	1.29 ^a^	7,222	15.31 ^a^	7,222	4.74 ^a^	7,222	20.33 ^a^	9,426	4.39 ^a^
Toddlers	8,599	4.04 ^ac^	8,599	1.12 ^a^	8,599	11.33 ^ad^	8,599	13.39 ^ac^	8,599	26.20 ^ac^	17,801	8.41 ^ac^
Preschoolers	12,778	3.97 ^ac^	12,778	1.32 ^a^	12,778	6.04 ^df^	12,778	22.77 ^ace^	12,778	23.99 ^acf^	35,738	13.20 ^ace^
School-aged children	8,491	5.05 ^aceg^	8,491	1.96 ^aceg^	8,491	2.46 ^bdfh^	8,491	24.00 ^aceg^	8,491	16.31 ^adfh^	27,912	20.85 ^aceg^
Adolescents	649	3.54 ^a^	649	0.92	649	2.31 ^bdfh^	649	8.63 ^acfhi^	649	14.48 ^adfh^	2,543	12.33 ^acei^
*χ* ^2^		127.091		35.579		1,089.209		1,702.868		448.432		1,328.325
*p*-value		< 0.001		< 0.001		< 0.001		< 0.001		< 0.001		< 0.001

a Significantly increased compared to newborn group.

b Significantly decreased compared to newborn group.

c Significantly increased compared to infant group.

d Significantly decreased compared to infant group.

e Significantly increased compared to toddler group.

f Significantly decreased compared to toddler group.

g Significantly increased compared to preschooler group.

h Significantly decreased compared to preschooler group.

i Significantly decreased compared to school-aged group.

### Comparison of the prevalence of six respiratory pathogens in different seasons and months among children

The prevalence of Flu A, Flu B, and RSV among children was higher in spring and winter, and significantly higher than in summer and autumn (*p* < 0.001). Among them, the prevalence of Flu A was higher in winter than in spring (7.70% vs 3.91%, *p* < 0.05), while there was no significant difference in the prevalence of Flu B and RSV between winter and spring (2.82% vs 2.77% for Flu B and 15.34% vs 15.42% for RSV, both *p*>0.05). The prevalence of MP and RV among children was significantly higher in spring and summer than in autumn and winter (*p* < 0.001). Among them, the prevalence of MP was higher in spring than in summer (15.48% vs 12.41%, *p* < 0.05) as well as higher in winter than in autumn (10.64% vs 8.57%, *p* < 0.05), while no significant difference was observed in the prevalence of RV between spring and summer (23.16% vs 23.19%, *p*>0.05) or between winter and autumn (19.20% vs 20.21%, *p*>0.05). The prevalence of ADV among children was significantly higher in autumn and summer than in winter and spring (*p* < 0.001), with that in autumn higher than in summer (23.01% vs 21.59%, *p* < 0.05) and that in winter higher than in spring (13.53% vs 7.44%, *p* < 0.05). The comparison of the prevalence of six respiratory pathogens in different seasons among children is reported in **Table 4**.

**Table 4:** Comparison of prevalence of Flu A, Flu B, RSV, ADV, RV and MP in different seasons among children.

Seasons	Flu A		Flu B		RSV		ADV		RV		MP	
	n	Prevalence (%)	n	Prevalence (%)	n	Prevalence (%)	n	Prevalence (%)	n	Prevalence (%)	n	Prevalence (%)
Spring	8,471	3.91	8,471	2.77	8,471	15.42	8,471	7.44	8,471	23.16	8,471	15.48
Summer	11,286	1.35 ^b^	11,286	0.04 ^b^	11,286	0.80 ^b^	11,286	21.59 ^a^	11,286	23.19	11,286	12.41 ^b^
Autumn	8,891	2.09 ^bc^	8,891	0.00 ^bd^	8,891	1.89 ^bc^	8,891	23.01 ^ac^	8,891	20.21 ^bd^	8,891	8.57 ^bd^
Winter	10,542	7.70 ^ace^	10,542	2.82 ^ce^	10,542	15.34 ^ce^	10,542	13.53 ^adf^	10,542	19.20 ^bd^	10,542	10.64 ^bde^
*χ* ^2^		699.877		557.220		2,615.577		1,048.390		74.780		217.707
*p*-value		< 0.001		< 0.001		< 0.001		< 0.001		< 0.001		< 0.001

a Significantly increased compared to spring.

b Significantly decreased compared to spring.

c Significantly increased compared to summer.

d Significantly decreased compared to summer.

e Significantly increased compared to autumn.

f Significantly decreased compared to autumn.

**Figure 3:**
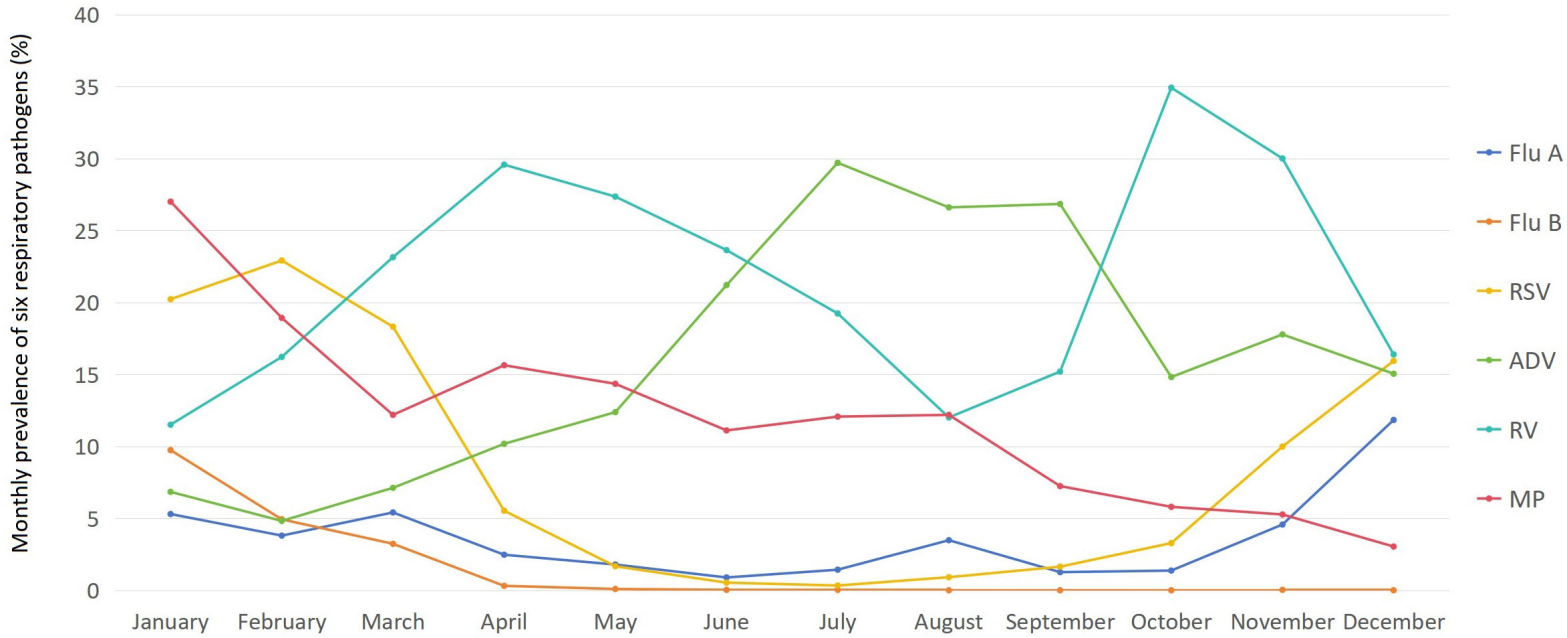
Monthly prevalence of six respiratory pathogens from January to December 2024 (%).

There were significant differences in the monthly prevalence of each of the six pathogens among children (*χ*^2^ ranging from 1101.382 to 3467.756, with all *p* < 0.001). The prevalence of Flu A among children was the highest in December (11.84%), followed by March (5.41%) and January (5.30%), while it remained at a relatively low level below 2% from May to July and September to October (ranging from 0.89% to 1.79%). The prevalence of Flu B among children was the highest in January (9.74%), followed by February (4.93%) and March (3.23%), but it stayed at an extremely low level below 0.1% from May to December (ranging from 0.00% to 0.09%). The prevalence of RSV among children was the highest in February (22.92%), followed by January (20.23%) and March (18.32%), while it remained at a relatively low level below 2% from May to September (ranging from 0.33% to 1.67%). The prevalence of ADV among children was generally high, with only January to March showing levels below 10% (ranging from 4.82% to 7.12%), while all other months saw levels above 10%, and from June to September, levels even exceeded 20% (ranging from 21.21% to 29.71%). The prevalence of RV among children exceeded 10% in all months (the lowest of 11.51% in January), with October and November reaching over 30% (34.39% in October and 30.01% in November), and from March to June standing between 20% and 30% (ranging from 23.15% to 29.58%). The prevalence of MP among children was generally high, peaking in January at 27.01%, with levels remaining above 10% from February to August (ranging from 11.11% to 18.93%), while staying above 5% from September to November (ranging from 5.27% to 7.24%) and only December seeing a level below 5% (3.04%). The monthly prevalence of six respiratory pathogens among children is shown in **Figure 3**.

### Comparison of the prevalence of six respiratory pathogens in different clinical diagnoses among children

The prevalence of Flu A and Flu B among children was the highest in the SRLT group (10.27% for Flu A and 4.11% for Flu B) and the lowest in the CRDs group (0.76% for Flu A and 0.38% for Flu B), with the remaining clinical diagnosis groups—ALRTIs, AURTIs and NRDs—showing intermediate values. Specifically, for Flu A, the prevalence decreased in the following order: AURTIs (4.74%), NRDs (3.35%), and ALRTIs (3.31%). For Flu B, the order of decreasing prevalence was: NRDs (1.42%), ALRTIs (1.34%), and AURTIs (1.05%). There were significant differences in prevalence among these five groups (all *p* < 0.001). The prevalence of Flu A in the AURTIs group was significantly higher than that in the ALRTIs group (*p* < 0.05). However, there was no significant difference in the prevalence of Flu B between the AURTIs group and the ALRTIs group (*p*>0.05). The prevalence of RSV and MP among children was the highest in the ALRTIs group (10.79% for RSV and 15.41% for MP) and the lowest in the AURTIs group (2.10% for RSV and 3.99% for MP), with the remaining clinical diagnosis groups—CRDs, NRDs and SRLT—showing intermediate values. Specifically, for RSV, the prevalence decreased in the following order: SRLT (7.36%), CRDs (5.70%), and NRDs (3.09%). For MP, the order of decreasing prevalence was: CRDs (9.13%), SRLT (8.98%), and NRDs (4.29%). There were significant differences in prevalence among these five groups (all *p* < 0.001).

The prevalence of RV among children was the highest in the CRDs group (44.87%) and the lowest in the AURTIs group (14.99%), with the ALRTIs (24.02%), SRLT (18.73%), and NRDs (16.63%) showing intermediate values in descending order; there were significant differences in prevalence among these five groups (*p* < 0.001). The prevalence of ADV among children was the highest in the AURTIs group (38.09%) and the lowest in the CRDs group (6.84%), with the SRLT (19.93%), NRDs (12.68%), and ALRTIs (11.21%) showing intermediate values in descending order; there were significant differences in prevalence among these five groups (*p* < 0.001). The comparison of the prevalence of six respiratory pathogens in different clinical diagnoses among children is reported in **Table 5**.

**Table 5:** Comparison of prevalence of Flu A, Flu B, RSV, ADV, RV and MP in different clinical diagnoses among children.

Clinical diagnoses	Flu A		Flu B		RSV		ADV		RV		MP	
	n	Prevalence (%)	n	Prevalence (%)	n	Prevalence (%)	n	Prevalence (%)	n	Prevalence (%)	n	Prevalence (%)
ALRTIs	25,795	3.31	25,795	1.34	25,795	10.79	25,795	11.21	25,795	24.02	25,795	15.41
AURTIs	7,398	4.74 ^a^	7,398	1.05	7,398	2.10 ^b^	7,398	38.09 ^a^	7,398	14.99 ^b^	7,398	3.99 ^b^
CRDs	263	0.76 ^bd^	263	0.38	263	5.70 ^bc^	263	6.84 ^bd^	263	44.87 ^ac^	263	9.13 ^bc^
NRDs	4,565	3.35 ^de^	4,565	1.42	4,565	3.09 ^bd^	4,565	12.68 ^ade^	4,565	16.63 ^bcf^	4,565	4.29 ^bf^
SRLT	1,169	10.27 ^aceg^	1,169	4.11 ^aceg^	1,169	7.36 ^bcg^	1,169	19.93 ^adeg^	1,169	18.73 ^bcf^	1,169	8.98 ^bcg^
*χ* ^2^		178.402		72.414		765.063		3,075.091		438.008		1.020.650
*p*-value		< 0.001		< 0.001		< 0.001		< 0.001		< 0.001		< 0.001

a Significantly increased compared to ALRTIs group.

b Significantly decreased compared to ALRTIs group.

c Significantly increased compared to AURTIs group.

d Significantly decreased compared to AURTIs group.

e Significantly increased compared to CRDs group.

f Significantly decreased compared to CRDs group.

g Significantly increased compared to NRDs group.

h Significantly decreased compared to NRDs group.

### Associations between Flu A, Flu B, RSV, ADV, RV and MP positivity and variables of gender, age, seasons, clinical diagnoses and medical settings by univariate logistic regression analysis

Univariate logistic regression analysis showed that Flu A, Flu B, RSV, ADV, RV, and MP positivity were significantly associated with medical settings (inpatient vs outpatient, all *p* < 0.001). Flu B and RV positivity were significantly associated with gender (females vs males, both *p* < 0.01), but Flu A, RSV, ADV, and MP were not significantly associated with gender (females vs males, all *p*>0.05). Compared to the newborns, Flu A, ADV, RV, and MP positivity were significantly associated with the infants, toddlers, preschoolers, school-aged children, and adolescents (all *p* < 0.05). Flu B positivity was significantly associated with the infants, toddlers, preschoolers, and school-aged children (all *p* < 0.05), but not with the adolescents (*p* = 0.241). RSV positivity was significantly associated with the infants, toddlers, school-aged children, and adolescents (all *p* < 0.05), but not with the preschoolers (*p* = 0.073). Compared to spring, Flu A, ADV, and MP positivity were significantly associated with summer, autumn and winter (all *p* < 0.001). Flu B positivity was significantly associated with summer (*p* < 0.001), but not with autumn and winter (both *p*>0.05). RSV positivity was significantly associated with summer and autumn (both *p* < 0.001), but not with winter (*p* = 0.881). RV positivity was significantly associated with autumn and winter (both *p* < 0.001), but not with summer (*p* = 0.965). Compared to ALRTIs group, RSV, ADV, RV, and MP positivity were significantly associated with AURTIs, CRDs, NRDs and SRLT groups (all *p* < 0.05). Flu A positivity was significantly associated with AURTIs, CRDs and SRLT groups (all *p* < 0.05), but not with NRDs group (*p* = 0.898). Flu B positivity was significantly associated with SRLT group (*p* < 0.001), but not with AURTIs, CRDs, and NRDs groups (all *p*>0.05). The associations between Flu A, Flu B, RSV, ADV, RV and MP positivity and variables of gender, age, seasons, clinical diagnoses and medical settings among children are reported in **Table 6** and its (Continued).

**Table 6:** Univariate logistic regression analysis of the association between Flu A, Flu B, RSV, ADV, RV and MP positivity and variables of gender, age, seasons, clinical diagnoses and medical settings among children.

Variables	Flu A				Flu B				RSV			
	Odds ratio	95% CI	Wald	*p*-value	Odds ratio	95% CI	Wald	*p*-value	Odds ratio	95% CI	Wald	*p*-value
Gender												
Males	1	–	–	–	1	–	–	–	1	–	–	–
Female	1.045	0.942–1.160	0.692	0.405	0.763	0.640–0.909	9.131	0.003	0.931	0.865–1.001	3.707	0.054
Medical settings												
Outpatient	1	–	–	–	1	–	–	–	1	–	–	–
Inpatient	0.349	0.313–0.388	371.989	<0.001	0.518	0.431–0.622	49.728	<0.001	1.514	1.367–1.678	63.144	<0.001
Age groups												
Newborns	1	–	–	–	1	–	–	–	1	–	–	–
Infants	5.771	2.551–13.053	17.716	<0.001	2.691	1.246–5.814	6.345	0.012	2.318	1.882–2.856	62.368	<0.001
Toddlers	10.127	4.510–22.724	31.464	<0.001	2.329	1.079–5.027	4.639	0.031	1.637	1.328–2.019	21.288	<0.001
Preschoolers	9.95	4.441–22.294	31.161	<0.001	2.765	1.296–5.900	6.916	0.009	0.824	0.667–1.018	3.207	0.073
School-aged children	12.815	5.714–28.741	38.311	<0.001	4.113	1.927–8.781	13.361	<0.001	0.323	0.254–0.412	83.948	<0.001
Adolescents	8.849	3.585–21.837	22.376	<0.001	1.925	0.644–5.750	1.376	0.241	0.303	0.175–0.525	18.13	<0.001
Seasons												
Spring	1	–	–	–	1	–	–	–	1	–	–	–
Summer	0.336	0.276–0.408	121.4	<0.001	0.016	0.006–0.038	84.832	<0.001	0.044	0.036–0.055	804.769	<0.001
Autumn	0.525	0.438–0.630	47.951	<0.001	0	/	0.002	0.967	0.106	0.090–0.124	724.486	<0.001
Winter	2.052	1.800–2.340	115.423	<0.001	1.016	0.854–1.209	0.032	0.858	0.994	0.918–1.076	0.022	0.881
Clinical diagnoses												
ALRTIs	1	–	–	–	1	–	–	–	1	–	–	–
AURTIs	1.435	1.280–1.650	33.22	<0.001	0.786	0.614–1.006	3.646	0.056	0.177	0.150–0.208	429.156	<0.001
CRDs	0.224	0.056–0.900	4.445	0.035	0.282	0.039–2.012	1.596	0.207	0.5	0.296–0.843	6.76	0.009
NRDs	1.012	0.849–1.205	0.017	0.898	1.066	0.816–1.392	0.217	0.641	0.263	0.222–0.313	230.471	<0.001
SRLT	3.337	2.730–4.079	138.343	<0.001	3.159	2.322–4.297	53.636	<0.001	0.656	0.525–0.820	13.686	<0.001
Gender												
Males	1	–	–	–	1	–	–	–	1	–	–	–
Female	1.001	0.945–1.056	0.001	0.973	0.899	0.856–0.944	18.277	<0.001	1.062	0.998–1.129	3.609	0.057
Medical settings												
Outpatient	1	–	–	–	1	–	–	–	1	–	–	–
Inpatient	0.344	0.324–0.364	1,290.37	<0.001	1.572	1.471–1.680	178.111	<0.001	1.32	1.215–1.434	43.152	<0.001
Age groups												
Newborns	1	–	–	–	1	–	–	–	1	–	–	–
Infants	1.628	1.179–2.247	8.778	0.003	2.487	2.065–2.996	92.003	<0.001	3.46	2.170–5.516	27.209	<0.001
Toddlers	5.06	3.712–6.897	105.289	<0.001	3.461	2.881–4.158	175.779	<0.001	6.919	4.372–10.949	68.221	<0.001
Preschoolers	9.652	7.106–13.110	210.556	<0.001	3.076	2.565–3.689	146.875	<0.001	11.464	7.270–18.078	110.154	<0.001
School-aged children	10.341	7.604–14.064	221.748	<0.001	1.9	1.577–2.289	45.621	<0.001	19.849	12.585–31.305	165.217	<0.001
Adolescents	3.092	2.055–4.654	29.29	<0.001	1.651	1.246–2.188	12.2	<0.001	10.597	6.366–17.638	82.443	<0.001
Seasons												
Spring	1	–	–	–	1	–	–	–	1	–	–	–
Summer	3.428	3.124–3.761	678.001	<0.001	1.001	0.937–1.071	0.002	0.965	0.774	0.714–0.840	38.194	<0.001
Autumn	3.72	3.383–4.091	734.562	<0.001	0.84	0.782–0.903	22.226	<0.001	0.512	0.466–0.563	191.748	<0.001
Winter	1.947	1.764–2.148	175.739	<0.001	0.788	0.735–0.845	44.391	<0.001	0.651	0.597–0.709	97.324	<0.001
Clinical diagnoses												
ALRTIs	1	–	–	–	1	–	–	–	1	–	–	–
AURTIs	4.875	4.587–5.180	2,606.22	<0.001	0.558	0.520–0.598	267.474	<0.001	0.228	0.202–0.257	571.278	<0.001
CRDs	0.582	0.360–0.941	4.879	0.027	2.575	2.016–3.288	57.395	<0.001	0.551	0.362–0.840	7.695	0.006
NRDs	1.151	1.046–1.266	8.334	0.004	0.631	0.581–0.686	118.314	<0.001	0.246	0.213–0.285	349.073	<0.001
SRLT	1.972	1.700–2.288	80.215	<0.001	0.729	0.628–0.847	17.081	<0.001	0.542	0.442–0.664	34.957	<0.001

### Multivariate logistic regression analysis results for associations between six respiratory pathogens positivity and associated variables (stepwise forward Wald)

**Table 7** and its (Continued) present the results of stepwise multivariate logistic regression analysis after adjustment for potential confounders. Flu B positivity was independently associated with gender (females vs males, OR: 0.742, 95% CI: 0.621–0.887, *p* = 0.001). Similarly, RV positivity was also independently associated with gender (females vs males, OR: 0.918, 95% CI: 0.873–0.964, *p* < 0.001). However, no association was observed between Flu A, ADV, RSV, or MP positivity and gender (females vs males). Flu A, Flu B, ADV, RV, and MP positivity were independently associated with medical settings (inpatient vs outpatient, all *p* < 0.01), but RSV positivity was not associated with medical settings. Compared with the newborns, Flu A, RV, and MP positivity were independently associated with the infants, toddlers, preschoolers, school-aged children, and adolescents (all *p* < 0.001), with all these age groups showing higher ORs. The highest OR was observed in the school-aged children for the Flu A model (OR: 9.410, 95% CI: 4.183–21.165), the toddlers for the RV model (OR: 4.003, 95% CI: 3.328–4.814), and the school-aged children for the MP model (OR: 31.875, 95% CI: 20.179–50.351), respectively. Flu B positivity was independently associated with the infants, preschoolers, and school-aged children (all *p* < 0.05), with all these groups showing higher ORs and the school-aged children having the highest (OR: 3.905, 95% CI: 1.819–8.383). ADV positivity was independently associated with the toddlers, preschoolers, school-aged children, and adolescents (all *p* < 0.01), with all these groups showing higher ORs and the preschoolers having the highest (OR: 6.361, 95% CI: 4.673–8.659). RSV positivity was independently associated with the infants, toddlers, school-aged children, and adolescents (all *p* < 0.01): higher ORs were observed in the infants and toddlers, while lower ORs were seen in the school-aged children and adolescents.

**Table 7:** Multivariate logistic regression (Stepwise Forward Wald) for Flu A, Flu B, RSV, ADV, RV and MP positivity among 39,190 children aged 0–18.

Variables	ADV				RV				MP			
	Odds ratio	95% CI	Wald	*p*-value	Odds ratio	95% CI	Wald	*p*-value	Odds ratio	95% CI	Wald	*p*-value
Gender												
Males	1	–	–	–	1	–	–	–	1	–	–	–
Female	/	/	/	/	0.742	0.621–0.887	10.805	0.001	/	/	/	/
Medical settings												
Outpatient	1	–	–	–	1	–	–	–	1	–	–	–
Inpatient	0.378	0.332–0.431	214.677	<0.001	0.442	0.365–0.534	70.737	<0.001	/	/	/	/
Age groups												
Newborns	1	–	–	–	1	–	–	–	1	–	–	–
Infants	5.443	2.404–12.326	16.506	<0.001	2.529	1.168–5.476	5.54	0.019	2.606	2.102–3.232	76.075	<0.001
Toddlers	8.348	3.709–18.790	26.283	<0.001	2.167	1.000–4.700	3.836	0.051	2.261	1.821–2.808	54.487	<0.001
Preschoolers	8.05	3.585–18.077	25.538	<0.001	2.716	1.288–5.917	6.814	0.009	1.069	0.860–1.330	0.365	0.546
School-aged children	9.41	4.183–21.165	29.379	<0.001	3.905	1.819–8.383	12.218	<0.001	0.431	0.337–0.553	44.147	<0.001
Adolescents	6.577	2.650–16.321	16.496	<0.001	1.728	0.575–5.191	0.949	0.33	0.438	0.251–0.766	8.369	0.004
Seasons												
Spring	1	–	–	–	1	–	–	–	1	–	–	–
Summer	0.267	0.219–0.325	172.384	<0.001	0.013	0.005–0.032	90.916	<0.001	0.052	0.042–0.064	715.576	<0.001
Autumn	0.426	0.353–0.514	79.878	<0.001	0	/	0.002	0.996	0.124	0.105–0.146	612.287	<0.001
Winter	1.804	1.579–2.062	74.908	<0.001	0.905	0.758–1.080	1.22	0.269	1.021	0.942–1.017	0.364	0.607
Clinical diagnoses												
ALRTIs	1	–	–	–	1	–	–	–	1	–	–	–
AURTIs	1.225	1.062–1.413	7.715	0.005	/	/	/	/	0.297	0.251–0.352	197.505	<0.001
CRDs	0.263	0.065–1.062	3.52	0.061	/	/	/	/	0.737	0.426–1.274	1.198	0.274
NRDs	1.173	0.979–1.406	2.999	0.083	/	/	/	/	0.329	0.276–0.393	150.759	<0.001
SRLT	1.2	0.956–1.507	2.466	0.116	/	/	/	/	0.808	0.639–1.022	3.154	0.076
Gender												
Males	1	–	–	–	1	–	–	–	1	–	–	–
Female	/	/	/	/	0.918	0.873–0.964	11.564	<0.001	/	/	/	/
Medical settings												
Outpatient	1	–	–	–	1	–	–	–	1	–	–	–
Inpatient	0.631	0.588–0.677	161.676	<0.001	1.513	1.399–1.635	108.925	<0.001	1.137	1.033–1.252	6.869	0.009
Age groups												
Newborns	1	–	–	–	1	–	–	–	1	–	–	–
Infants	1.33	0.962–1.839	2.97	0.085	2.549	2.115–3.072	96.584	<0.001	3.528	2.212–5.629	27.988	<0.001
Toddlers	3.099	2.268–4.237	50.329	<0.001	4.003	3.328–4.814	216.775	<0.001	8.931	5.638–14.147	87.023	<0.001
Preschoolers	6.361	4.673–8.659	138.283	<0.001	3.524	2.936–4.231	182.498	<0.001	14.956	9.474–23.610	134.839	<0.001
School-aged children	6.209	4.553–8.467	133.08	<0.001	2.315	1.919–2.793	76.862	<0.001	31.875	20.179–50.351	220.24	<0.001
Adolescents	2.054	1.354–3.115	11.465	0.001	2.116	1.593–2.811	26.76	<0.001	20.081	11.992–33.626	130.058	<0.001
Seasons												
Spring	1	–	–	–	1	–	–	–	1	–	–	–
Summer	2.509	2.278–2.763	349.39	<0.001	1.067	0.997–1.143	3.489	0.062	0.753	0.690–0.821	41.658	<0.001
Autumn	2.7	2.444–2.983	381.155	<0.001	0.924	0.858–0.995	4.347	0.037	0.526	0.509–0.621	128.193	<0.001
Winter	1.637	1.479–1.813	89.663	<0.001	0.809	0.753–0.868	34.268	<0.001	0.565	0.516–0.618	154.484	<0.001
Clinical diagnoses												
ALRTIs	1	–	–	–	1	–	–	–	1	–	–	–
AURTIs	3.25	3.036–3.480	1,144.24	<0.001	0.599	0.555–0.646	177.809	<0.001	0.183	0.161–0.208	675.794	<0.001
CRDs	0.449	0.277–0.729	10.505	0.001	2.374	1.853–3.041	46.819	<0.001	0.377	0.246–0.578	20.031	<0.001
NRDs	1.085	0.983–1.198	2.642	0.104	0.625	0.574–0.680	118.349	<0.001	0.18	0.155–0.209	498.052	<0.001
SRLT	1.061	0.902–1.248	0.515	0.473	1.013	0.858–1.196	0.023	0.879	0.441	0.353–0.551	51.955	<0.001

Based on the results of univariate analysis, five variables including gender, age, season, clinical diagnosis, and medical setting were included in the initial model to analyze the associations between the positivity of six pathogens and associated variables. Among these variables, gender was excluded from the Flu A, ADV and MP models, respectively; clinical diagnosis was excluded from the Flu B model; gender and medical setting were excluded from the RSV model (multivariate logistic regression analysis using the Stepwise Forward Wald method).

Compared with spring, Flu A, ADV, and MP positivity were independently associated with summer, autumn, and winter (all *p* < 0.001). In the ADV model, ORs were elevated in summer, autumn, and winter, peaking in autumn (OR: 2.700, 95% CI: 2.444–2.983). Conversely, the MP model showed reduced ORs during these seasons, with the lowest value in autumn (OR: 0.526, 95% CI: 0.509–0.621), while the Flu A model had a higher OR only in winter and lower ones in summer and autumn. For the Flu B model, season (summer vs spring, OR: 0.013, 95% CI: 0.005–0.032, *p* < 0.001) was independently associated with Flu B positivity. However, there were no significant differences in Flu B positivity between autumn and spring (*p* = 0.996) or between winter and spring (*p* = 0.269). For the RSV model, seasons (summer vs spring, OR: 0.052, 95% CI: 0.042–0.064; autumn vs spring, OR: 0.124, 95% CI: 0.105–0.146, both *p* < 0.001) were independently associated with RSV positivity. However, there was no significant difference in RSV positivity between winter and spring (*p* = 0.607). For the RV model, seasons (autumn vs spring, OR: 0.924, 95% CI: 0.858–0.995; winter vs spring, OR: 0.809, 95% CI: 0.753–0.868, both *p* < 0.05) were independently associated with RV positivity. However, there was no significant difference in RV positivity between summer and spring (*p* = 0.062).

In five different clinical diagnostic groups, clinical diagnosis was excluded in the Flu B model. For the Flu A model, compared with the ALRTIs group, Flu A positivity was independently associated with a higher OR in the AURTIs group (OR: 1.225, 95% CI: 1.062–1.413, *p* = 0.005), but not significantly associated with the CRDs, NRDs, and SRLT groups (all *p*>0.05). For the RSV model, compared with the ALRTIs group, RSV positivity was independently associated with lower ORs in the AURTIs group (OR: 0.297, 95% CI: 0.251–0.352, *p* < 0.001) as well as the NRDs group (OR: 0.329, 95% CI: 0.276–0.393, *p* < 0.001), but not significantly associated with the CRDs and SRLT groups (both *p*>0.05). For the ADV model, compared with the ALRTIs group, ADV positivity was independently associated with a higher OR in the AURTIs group (OR: 3.250, 95% CI: 3.036–3.480, *p* < 0.001) and a lower OR in the CRDs group (OR: 0.449, 95% CI: 0.277–0.729, *p* = 0.001), but not significantly associated with the NRDs and SRLT groups (both *p*>0.05). For the RV model, compared with the ALRTIs group, RV positivity was independently associated with a higher OR in the CRDs group (OR: 2.374, 95% CI: 1.853–3.041, *p* < 0.001) and lower ORs in the AURTIs group (OR: 0.599, 95% CI: 0.555–0.646, *p* < 0.001) as well as in the NRDs group (OR: 0.625, 95% CI: 0.574–0.680, *p* < 0.001), but not significantly associated with the SRLT group (*p* = 0.879). For the MP model, compared with the ALRTIs group, MP positivity was independently associated with lower ORs in the AURTIs, CRDs, NRDs and SRLT groups, with the lowest OR in the NRDs group (OR: 0.180, 95% CI: 0.155–0.209, *p* < 0.001).

## Discussion

RTIs are common respiratory diseases among children and are the main cause of morbidity, hospitalization and death among children and cause economic losses to both families and society, with pathogen distribution patterns varying by region, age, and season (Zhu et al., 2021; Neumann and Kawaoka, 2022; GBD 2021 Lower Respiratory Infections and Antimicrobial Resistance Collaborators, 2024). Due to overlapping infections involving different pathogens (including viruses and atypical pathogens) and the numerous similarities in their clinical manifestations, diagnosing the pathogens causing RTIs poses a significant challenge. Different pathogens typically require distinct treatment strategies: for instance, viral infections may call for antiviral drugs, symptomatic interventions, and other forms of supportive care, while infections caused by MP usually respond to specific antibiotics (Wahab et al., 2022; Georgakopoulou et al., 2024). However, their shared symptoms such as fever and cough make it difficult to distinguish them based solely on clinical manifestations, which underscores the need for accurate pathogen detection to guide targeted treatment.

A retrospective study on patients’ respiratory specimens in Tengzhou China, tested for Flu A, Flu B, RSV, ADV, RV, and MP between November 2023 and January 2024 by Ma H et al (Ma et al., 2025), showed that 47.4% were identified as single-pathogen infections, 10.9% as dual-pathogen infections, and 1.5% as infections involving three or more pathogens. Flu A was the most frequently detected pathogen (27.5%), followed by RSV (10.1%), ADV (9.7%), MP (4.6%), RV (4.5%), and Flu B (2.9%). Another retrospective study, analyzing the same six respiratory pathogens among children in Yongzhou, China, from June 2023 to May 2024, by Tang Z et al (Tang et al., 2025), reported an overall prevalence of 77.0%: 52.0% single-pathogen infections and 25.0% mixed infections. RV had the highest prevalence (32.4%), followed by MP (20.9%), ADV (19.2%), RSV (14.1%), Flu A (11.5%), and Flu B (8.3%).

Our study shows that more than half (55.53%) of the children were infected with at least one of the six respiratory pathogens. The overall prevalence of single infection, double co-infection, and triple co-infection was 48.19%, 7.09%, and 0.25%, respectively, with no quadruple, quintuple, or sextuple co-infections (i.e., 4, 5, or 6 pathogens) observed. The pathogen-specific prevalence from the highest to the lowest was as follows: RV (21.43%), ADV (16.69%), MP (11.73%), RSV (8.12%), Flu A (3.78%), and Flu B (1.37%) (*χ*^2^ = 12,221.187, *p* < 0.001). For single infections, the pathogens with the highest prevalence were RV (16.32%), ADV (12.98%), and MP (8.76%), while those with the lowest prevalence were Flu B (1.01%), Flu A (3.00%), and RSV (6.12%). For double co-infections, the combinations with the highest prevalence were ADV+RV (2.18%), RV+MP (1.48%), and RSV+RV (0.94%), whereas those with the lowest prevalence were Flu A+Flu B (0.01%), Flu B+ADV (0.05%), and Flu B+RV (0.05%). For triple co-infections, ADV+RV+MP (0.08%), RSV+ADV+RV (0.05%), and RSV+RV+MP (0.03%) showed the highest prevalence, in contrast to the three combinations including Flu A+Flu B+ADV, Flu A+RSV+MP, and Flu B+RSV+ADV, for which no cases were detected.

The overall prevalence of the six pathogens and the prevalence of single-pathogen infections in this study were both lower than those in the Tang Z et al. study but close to those in the Ma H et al. study. However, the prevalence of multiple infections in this study was lower than that in both the Ma H et al. (10.9% dual, 1.5% triple or more) and the Tang Z et al. studies (25.0% mixed). Notably, this study further delineated the prevalence hierarchy of specific double and triple co-infection combinations—for example, ADV+RV was the most common dual combination (2.18%), and ADV+RV+MP was the most frequent triple combination (0.08%). This distinction may stem from regional differences in dominant pathogen circulation: ADV and RV, the dominant pathogens of the most prevalent co-infection combinations in our study, had relatively higher overall prevalence locally (16.69% and 21.43%, respectively) compared with the Ma H et al. study (ADV: 9.7%, RV: 4.5%), potentially increasing their chance of co-infections.

Moreover, no cases of infections involving four or more pathogens were observed in the present study, which aligns with the lack of such reports in the Ma H et al. and the Tang Z et al. studies, suggesting that quadruple or higher co-infections may be rare in pediatric respiratory infections. In terms of specific pathogens, among the six pathogens in this study, Flu B had the lowest prevalence, followed by Flu A and RSV, while RV had the highest prevalence, which is consistent with the findings of Tang Z et al. However, our study found that ADV was the second highest and MP the third highest, which differs somewhat from the results of Tang Z et al. (where MP was second and ADV third). These differences indicate that the infection characteristics of respiratory pathogens—such as overall prevalence, types of single infections and multiple co-infections, and ranking of dominant pathogen prevalence—may be influenced by factors including study population, time frames, regional differences (e.g., geographic locations) and region-specific epidemic patterns.

In the present study, the prevalence of Flu B and RV was slightly higher among male children than among female children (1.53% vs 1.17% for Flu B, 22.22% vs 20.44% for RV, both *p* < 0.01). However, there was no significant difference in the prevalence of Flu A, RSV, ADV, and MP between male and female children (all *p*>0.05). After adjustment for potential confounders, multivariate logistic regression analysis showed that Flu B positivity was independently associated with gender (females vs males, OR: 0.742, 95% CI: 0.621–0.887, *p* = 0.001), and RV positivity was also independently associated with gender (females vs males, OR: 0.918, 95% CI: 0.873–0.964, *p* < 0.001). These results suggest that gender could be a contributing factor in the incidence of Flu B and RV infections among children: the higher prevalence of Flu B and RV in male children, along with the significant OR values from the regression analysis, indicates potential underlying biological or behavioral gender differences impacting susceptibility to these pathogens, though it’s important to note that sampling errors might influence this difference and affect the generalizability of our findings.

Gender impacts outcomes of various respiratory viral infections, with gender differences in pathogenesis evident across viruses like RSV and influenza throughout the life course. Notably, males tend to be more vulnerable to severe outcomes from such infections at younger and older ages (Ursin and Klein, 2021). These broader trends are supported by specific epidemiological data from Peer V et al (Peer et al., 2025), who conducted an 11-year analysis (2012–2022) of acute respiratory tract infections (ARTIs) among hospitalized cases at Sheba Medical Center, Israel. Focusing on pathogens including ADV, influenza viruses, RV, RSV, and others, their study revealed a male excess in infection rates for all viruses, with the most notable differences in the youngest age groups (<1 year and 1–4 years). Specifically, males were more likely to be positive for RV and influenza virus in infancy and toddlers, with RV positivity 40% and 25% higher, and influenza incidence rates 42% and 28% higher, respectively, which supports the gender-associated trend we observed for RV and Flu B (a type of influenza virus). These findings reinforce that male children may generally have a higher susceptibility to certain respiratory viruses such as RV and influenza viruses, particularly in toddlers.

Reports on differences in prevalence between outpatient and inpatient settings for these six respiratory pathogens are limited. A study by Nduaguba SO et al (Nduaguba et al., 2023), which assessed over 8 seasons and years from 2011 to 2019 among children under 5 years old, showed that the annual outpatient RSV infection rates for all age groups were higher than those of inpatient settings (1.29% vs 0.14%). While another study by Yu J et al (Yu et al., 2018). on ARTIs in 11 hospitals in North China from 2012 to 2015 showed that RSV was the most common virus in hospitalized children under 2 years old (33.3%), whereas influenza virus was the most common in outpatient/emergency patients across all age groups (22.7%). Our data showed that the prevalence of Flu A, Flu B, and ADV among children was higher in outpatient than in inpatient settings (all *p* < 0.001), while the prevalence of RSV, RV, and MP among children was lower in outpatient than in inpatient settings (all *p* < 0.001). Multivariate logistic regression analysis revealed that Flu A, Flu B, ADV, RV, and MP positivity were independently associated with medical settings (inpatient vs outpatient, all *p* < 0.01), but RSV positivity was not associated with medical settings.

These findings indicate that the association between the prevalence of respiratory pathogens and medical settings varies by pathogen, age, and region. For instance, Nduaguba SO et al. reported higher outpatient RSV rates among children under 5 in the U.S., while Yu J et al. observed higher RSV prevalence in hospitalized children under 2 in North China. Our results, which showed lower outpatient RSV prevalence and higher outpatient prevalence of Flu A and Flu B, are consistent with the findings of Yu J et al. that influenza viruses were the most common in outpatient/emergency patients across all age groups. Notably, despite unadjusted differences in RSV prevalence, our multivariate analysis found no association between RSV positivity and medical settings, implying that confounding factors (e.g., disease severity) may influence its prevalence distribution, and these findings warrant further investigation.

Numerous studies have indicated significant associations between the prevalence of respiratory pathogens and age among children (Jiang et al., 2021; Li et al., 2024; Wu et al., 2024). Zhu G et al (Zhu et al., 2021). showed that viral infection positivity varied significantly across age groups. ADV positivity was the highest among children aged 3–6 years (18.7%), while Flu A and Flu B were most prevalent in those over 6 years old (21.6% and 6.6%, respectively), and RSV positivity was the highest in infants under 1 year old (10.6%). Lv G et al (Lv et al., 2024). found that infants had a higher RSV infection rate (4.25%) than toddlers (1.98%) and older (preschoolers and school-aged) children (0.24%). Toddlers had a higher RV infection rate (6.34%) than infants (4.19%) and older children (2.82%). Flu A and MP infection rates were higher in older children (4.53% and 3.37%, respectively) than in infants (0.36% and 0.10%, respectively) and toddlers (1.65% and 0.16%, respectively). Flu B infection rates showed no significant age-associated difference.

More recently, a retrospective analysis of 11,538 children with RTIs between December 2022 and November 2023 showed that among children in different age groups, the older the children, the higher the infection rate of Flu A and MP, and the younger the children, the higher the positive rate of RSV, while the positive rate of ADV in children aged 3–6 years and > 6 years was higher than that in children aged 0–3 years (Wang et al., 2024). Another study analyzed 9,294 children aged 0–18 years with ARTIs symptoms from July 2023 to August 2024. This study revealed that the detection rates of pathogens varied among different age groups: MP was most common in school-aged children, while Flu A was more frequent in preschoolers (He et al., 2025).

In our study, the prevalence of Flu A, Flu B, ADV, and MP was the highest in school-aged children (5.05%, 1.96%, 24.00%, 20.85%) and the lowest in newborns (0.41%, 0.48%, 2.96%, 1.31%), significant differences were obtained in prevalence across six age groups (all *p* < 0.001); RSV was most prevalent in infants (15.31%) and least in adolescents (2.31%), with significant intergroup differences (*p* < 0.001); RV peaked in toddlers (26.20%) and was the lowest in newborns (9.30%; *p* < 0.001 across groups). Multivariate logistic regression (compared with newborns) showed Flu A, RV, and MP positivity were independently associated with all other age groups (higher ORs, all *p* < 0.001), with peak ORs in school-aged children for Flu A (OR: 9.410, 95% CI: 4.183–21.165) and MP (OR: 31.875, 95% CI: 20.179–50.351), and in toddlers for RV (OR: 4.003, 95% CI: 3.328–4.814); Flu B positivity associated with infants, preschoolers, and school-aged children (all *p* < 0.05), peaking in school-aged children (OR: 3.905, 95% CI: 1.819–8.383); ADV positivity associated with toddlers, preschoolers, school-aged children, and adolescents (all *p* < 0.01), with the highest OR in preschoolers (OR: 6.361, 95% CI: 4.673–8.659); RSV positivity associated with infants, toddlers, school-aged children, and adolescents (all *p* < 0.01), with higher ORs in infants/toddlers but lower in school-aged children/adolescents.

Our findings are largely consistent with previous studies, reinforcing the age-associated pattern of pediatric respiratory pathogen prevalence. Flu A and MP showed higher rates in older children across our data and prior research by Zhu G et al., Lv G et al., and the 2022–2023 retrospective analysis, with school-aged children most affected, supporting age-associated exposure or immune response differences; RSV, consistent with earlier work by Lv G et al. and the 2022–2023 analysis, was most prevalent in infants in our study, highlighting their vulnerability likely tied to immature immunity; ADV aligned with Zhu G et al. in showing peak rates in preschool/school-aged children, with our regression pinpointing preschoolers as the most strongly associated group; RV mirrored Lv G et al. in having the highest prevalence in toddlers, confirming this age as a key prevention target. Notably, our inclusion of newborns and adolescents adds nuance: newborns had the lowest rates (possibly due to maternal antibodies and/or their low probability of pathogen contact), while adolescents showed lower prevalence than school-aged children, reflecting shifting immunity and behavior. These consistent patterns underscore the need for age-specific strategies.

It is well-known that respiratory virus prevalence is significantly associated with seasons. Before SARS-CoV-2, Perez A et al (Perez et al., 2022). noted seasonal patterns, with influenza and RSV peaking in late autumn and winter. Chen Z et al (Chen et al., 2024). found COVID-19 significantly altered global seasonal influenza spread over three years. Similarly, Zhao X et al (Zhao et al., 2025). reported differing respiratory virus prevalence across pre-pandemic (2019), pandemic (2020–2022), and post-pandemic (2023) phases (*p* < 0.001): Flu A and Flu B predominated in winter-spring, RSV peaked in winter, and ADV had no distinct pattern. After SARS-CoV-2, Zhao C et al (Zhao et al., 2025). from the Respiratory Virus Global Epidemiology Network noted altered seasonal patterns and asynchronous resurgence with non-pharmaceutical interventions eased: their 92-site analysis revealed RV resurged first, followed by seasonal coronaviruses, parainfluenza viruses, RSV, ADV, metapneumovirus, and Flu A, with Flu B exhibiting the latest resurgence; the second resurgence was similar except Flu A caught up with metapneumovirus. This asynchrony reflects virus-specific adaptability to post-pandemic seasonal conditions.

Our study showed Flu A, Flu B, and RSV prevalence was higher in spring and winter than summer and autumn (*p* < 0.001); Flu A was more prevalent in winter than spring (7.70% vs 3.91%, *p* < 0.05), with no winter-spring differences for Flu B (2.82% vs 2.77%, *p*>0.05) or RSV (15.34% vs 15.42%, *p*>0.05). MP and RV were more common in spring and summer (*p* < 0.001); MP was higher in spring than summer (15.48% vs 12.41%, *p* < 0.05) and winter than autumn (10.64% vs 8.57%, *p* < 0.05), while RV showed no spring-summer (23.16% vs 23.19%, *p*>0.05) or winter-autumn (19.20% vs 20.21%, *p*>0.05) differences. ADV prevalence was significantly higher in autumn and summer than in winter and spring (*p* < 0.001), with that in autumn higher than in summer (23.01% vs 21.59%, *p* < 0.05) and that in winter higher than in spring (13.53% vs 7.44%, *p* < 0.05). Multivariate regression (compared with spring) showed Flu A, ADV, and MP were independently associated with summer, autumn, and winter (all *p* < 0.001): ADV had the highest OR in autumn (OR: 2.700, 95% CI: 2.444–2.983); MP had the lowest OR in autumn (OR: 0.526, 95% CI: 0.509–0.621); Flu A had higher OR in winter but lower in summer/autumn. Flu B associated with summer (OR: 0.013, *p* < 0.001), not autumn and winter. RSV associated with summer (OR: 0.052) and autumn (OR: 0.124, both *p* < 0.001), not winter. RV associated with autumn (OR: 0.924) and winter (OR: 0.809, both *p* < 0.05), not summer.

Our findings align with and extend prior observations. Consistent with Perez A et al., who noted pre-pandemic influenza and RSV peaked in late autumn and winter, our data showed Flu A, Flu B, and RSV predominated in winter and spring. However, we further found that RSV maintained high levels across both seasons rather than peaking strictly in winter, and Flu A was more prevalent in winter than spring. In line with Zhao X et al., who reported differing prevalence across pre-pandemic, pandemic, and post-pandemic phases, our results confirmed the winter-spring dominance of Flu A and Flu B but added refinements: Flu B showed no significant difference between winter and spring, and ADV, which was previously thought to have no distinct pattern, exhibited a clear autumn-summer predominance. This supports Zhao C et al. observation of post-pandemic virus-specific adaptability. Consistent with Zhao C et al., who identified RV as the first to resurge post-pandemic, our study found RV was most prevalent in spring and summer. MP spring-summer peak reflected stable seasonal trends, with multivariate ORs such as ADV strongest association with autumn and Flu A with winter further reinforcing these virus-specific seasonal associations.

We found that in addition to seasonal variations of six pathogens, significant monthly differences were observed in their prevalence. Flu A peaked in December (11.84%), followed by March (5.41%) and January (5.30%), with low levels (<2%) from May–July and September–October. Flu B was the highest in January (9.74%), then in February (4.93%) and March (3.23%), but extremely low (<0.1%) from May–December. RSV peaked in February (22.92%), January (20.23%), and March (18.32%), with low levels (<2%) from May–September. ADV was generally high: <10% only in January–March, >20% in June–September. RV exceeded 10% monthly (the lowest prevalence 11.51% in January), with >30% in October–November and 20–30% in March–June. MP was high overall, peaking in January (27.01%), >10% from February–August, >5% from September–November, and <5% only in December (3.04%). These monthly prevalence characteristics further reflect the potential association between China’s seasonal weather conditions (e.g., temperature, humidity) and pathogen transmission, laying a foundation for subsequent analysis of seasonal patterns.

Our analysis identifies the observed monthly prevalence differences as re-emerging seasonal patterns of these pathogens in Chengdu, Southwest China—a subtropical humid region. Notably, the December peak of Flu A, January-February peaks of RSV, and their sustained high prevalence in spring directly mirror this environment-driven seasonality. These trends align with the well-documented seasonality of respiratory viruses, shaped by environmental drivers (e.g., temperature, humidity) and their combined effects on virus stability, transmission, and host immune responses (Moriyama et al., 2020). For instance, the prominent winter peaks of influenza and RSV reflect their enhanced transmission under cold conditions: experimental evidence confirms influenza aerosol spread is favored by low temperature (e.g., 5 °C) and low relative humidity (e.g., 20%) (Lowen et al., 2007), while low absolute humidity further enhances the stability and airborne spread of influenza viruses (Shaman and Kohn, 2009). For RSV, its circulation correlates positively with rainfall and relative humidity and negatively with temperature in subtropical settings, consistent with Chengdu’s climate (Thongpan et al., 2020). The summer-autumn predominance of ADV and spring-summer peaks of RV/MP may relate to Chengdu’s subtropical environmental features, though further research is needed to confirm drivers. Thus, our monthly prevalence data validate the association between Chengdu’s seasonal weather and pathogen activity, supporting environmental drivers in regional viral seasonality and aligning with global influenza/RSV findings.

Beyond patterns associated with gender, medical settings, age, and seasons, exploring variations in these respiratory pathogens across different clinical diagnoses such as ALRTIs and AURTIs can further enhance our understanding of their clinical relevance and etiological associations. A previous systematic review and meta-analysis by Nair H et al (Nair et al., 2011), which included studies published from January 1, 1995, to October 31, 2010, identified influenza as a common pathogen in children with ALRTIs. It also noted that influenza imposes a significant burden on global health services. Another systematic review and meta-analysis by Pratt MTG et al (Pratt et al., 2022). covering studies published between January 1, 1995, and December 31, 2019, found that RSV (22.7%) and RV (22.1%) were the most common causes of pediatric pneumonia worldwide. A systematic analysis by Li Y et al (Li et al., 2022). showed that globally in 2019, among children aged 0–60 months, there were 33.0 million RSV-associated ALRTIs episodes, 3.6 million RSV-associated hospital admissions, 26,300 RSV-associated in-hospital deaths, and 101,400 total RSV-attributable deaths. Shieh WJ et al (Shieh, 2022). reported that ADV infection can cause both AURTIs, presenting with common cold-like symptoms such as rhinorrhea, fever, cough, and sore throat, and ALRTIs including bronchitis, bronchiolitis, and pneumonia. Similarly, Kumar S et al (Kumar and Kumar, 2023). found that MP infection can affect the upper respiratory tract, lower respiratory tract, or both.

We found that the prevalence of respiratory pathogens among children varied significantly across five clinical groups (ALRTIs, AURTIs, CRDs, NRDs, SRLT). Flu A and Flu B were most prevalent in the SRLT group (10.27%, 4.11%) and least in the CRDs group (0.76%, 0.38%), with the AURTIs group having higher Flu A prevalence than the ALRTIs group (*p* < 0.05). RSV and MP were most common in the ALRTIs group (10.79%, 15.41%) and least in the AURTIs group (2.10%, 3.99%). RV prevalence was the highest in the CRDs group (44.87%) and the lowest in the AURTIs group (14.99%), while ADV was most prevalent in the AURTIs group (38.09%) and least in the CRDs group (6.84%) (all *p* < 0.001 for inter-group differences). Multivariate logistic regression showed distinct pathogen associations across clinical groups compared with ALRTIs. The AURTIs group had higher Flu A (OR: 1.225) and ADV (OR: 3.250) positivity but lower RSV (OR: 0.297) and RV (OR: 0.599). The CRDs group featured higher RV (OR: 2.374) and lower ADV (OR: 0.449). NRDs had reduced RSV (OR: 0.329), RV (OR: 0.625), and notably MP (the lowest OR: 0.180), while SRLT only showed lower MP. Flu B had no group associations.

Our findings, integrated with prior research, reveal distinct pathogen distributions across the five clinical groups, and these patterns associate clinical diagnoses to pathogen-specific risks, aiding targeted management. Flu A and Flu B showed the highest prevalence in the SRLT group and the lowest in the CRDs group. Additionally, the AURTIs group had a significantly higher Flu A prevalence compared with the ALRTIs group (*p* < 0.05). Aligning with Pratt MTG et al. on RSV significance, RSV dominated in ALRTIs (10.79%) but was less common in AURTIs (OR: 0.297) and NRDs (OR: 0.329). ADV ability to cause both AURTIs and ALRTIs, as noted by Shieh WJ et al., was further supported by our findings showing its high prevalence in the AURTIs group (38.09%, OR: 3.250) and low prevalence in the CRDs group (6.84%, OR: 0.449). MP can affect both respiratory tracts as Kumar S et al. noted, which aligns with our finding of its presence across groups. However, our study further specifies its predominance in ALRTIs (15.41%) and lower rates in AURTIs (3.99%) and NRDs (OR: 0.180), whereas their research focuses on its dual-tract involvement. Notably, RV shows a distinct distribution across clinical groups, with the highest prevalence in CRDs (44.87%, OR: 2.374) and a lower but still substantial rate in AURTIs (14.99%). This further underscores these pathogen-clinical associations and supports tailored diagnostic and management strategies.

## Limitations

Firstly, limitations of single-site data: Data were derived from a single-site, resulting in a relatively narrow sample source. Future multi-center, large-scale studies are needed to improve the reliability of results and the generalizability of conclusions.

Secondly, limitations of the retrospective design: This study used a retrospective cross-sectional design, which may restrict us from establishing causal associations between pathogens and clinical outcomes—e.g., whether specific pathogens directly contribute to severe symptoms in certain age groups or diagnoses. Future prospective studies could help clarify such causal associations.

Thirdly, limited pathogen spectrum: Only six common respiratory pathogens (Flu A, Flu B, RSV, ADV, RV, MP) were included, excluding others like parainfluenza viruses, metapneumovirus, *Chlamydia pneumoniae*, *Streptococcus pneumoniae*, and *Klebsiella pneumoniae*. This may underestimate overall respiratory infection burden, overlook interactions between untested and tested pathogens, and fail to account for the potential role of *Klebsiella pneumoniae* (a common opportunistic bacterial pathogen associated with respiratory infections, especially in immunocompromised or hospitalized children) in pediatric RTIs. Expanding pathogen coverage to include *Klebsiella pneumoniae* and other untested pathogens is planned in follow-up research.

Fourthly, lack of detailed contextual data: Relevant factors influencing pathogen distribution—such as children’s vaccination status (e.g., influenza coverage), underlying conditions beyond defined clinical groups, and environmental exposures (e.g., daycare attendance)—were unanalyzed. Subsequent studies will include such contextual variables.

Finally, exclusion of coronaviruses: Our detection panel did not include coronaviruses, notably SARS-CoV-2. This precluded a dedicated assessment of their co-circulation and co-infection patterns with the six tested pathogens. Given the context of the COVID-19 pandemic, this represents a significant limitation, as it may lead to an incomplete understanding of the overall respiratory pathogen profile. Future studies should employ more comprehensive panels that incorporate coronaviruses.

## Conclusions

In the present study, using multiplex PCR, we found that over half of the children in the study population were infected with at least one of the six respiratory pathogens. The predominant pathogens were RV, ADV, and MP. While single infections constituted the majority, multiple co-infections were also observed. Among these, the most common dual and triple combinations were ADV+RV and ADV+RV+MP, respectively. Notably, the prevalence of these pathogens varied significantly across different groups. By age, school-aged children had the highest rates of Flu A, Flu B, ADV, and MP; infants were most susceptible to RSV; and toddlers had the highest RV prevalence. Seasonally, Flu A, Flu B, and RSV were more prevalent in spring and winter, MP and RV in spring and summer, and ADV in autumn and summer. Among clinical diagnoses, Flu A and Flu B were most common in SRLT, RSV and MP in ALRTIs, RV in CRDs, and ADV in AURTIs. Multiplex PCR enabled simultaneous detection of multiple respiratory pathogens in a single test, enhancing efficiency by reducing sample volume and testing time while facilitating accurate identification of both single and co-infections.

## Data Availability

The raw data supporting the conclusions of this article will be made available by the authors, without undue reservation.
